# *In Vitro* and *In Vivo* Antitumor Activity of a Novel Semisynthetic Derivative of Cucurbitacin B

**DOI:** 10.1371/journal.pone.0117794

**Published:** 2015-02-12

**Authors:** Izabella T. Silva, Annelise Carvalho, Karen L. Lang, Sabine E. Dudek, Dörthe Masemann, Fernando J. Durán, Miguel S. B. Caro, Ulf R. Rapp, Viktor Wixler, Eloir P. Schenkel, Cláudia M. O. Simões, Stephan Ludwig

**Affiliations:** 1 Department of Pharmaceutical Sciences, Universidade Federal de Santa Catarina, Florianópolis, SC, Brazil; 2 Department of Chemistry, Universidade Federal de Santa Catarina, Florianópolis, SC, Brazil; 3 Institute of Molecular Virology (IMV), Center of Molecular Biology of Inflammation (ZMBE), Westfaelische-Wilhelms-University, Muenster, Germany; 4 UMYMFOR, Department of Organic Chemistry, Universidad de Buenos Aires, Buenos Aires, Argentina; 5 Max Planck Institute for Heart and Lung Research, Bad Nauheim, Germany; 6 Cells in Motion, Cluster of Excellence, Westfaelische-Wilhelms-University, Muenster, Germany; 7 Interdisciplinary Center of Clinical Research (IZKF), Westfaelische-Wilhelms-University, Muenster, Germany; Institute of Biomedical Sciences, TAIWAN

## Abstract

Lung cancer is the most deadly type of cancer in humans, with non-small-cell lung cancer (NSCLC) being the most frequent and aggressive type of lung cancer showing high resistance to radiation and chemotherapy. Despite the outstanding progress made in anti-tumor therapy, discovering effective anti-tumor drugs is still a challenging task. Here we describe a new semisynthetic derivative of cucurbitacin B (DACE) as a potent inhibitor of NSCLC cell proliferation. DACE arrested the cell cycle of lung epithelial cells at the G2/M phase and induced cell apoptosis by interfering with EGFR activation and its downstream signaling, including AKT, ERK, and STAT3. Consistent with our *in vitro* studies, intraperitoneal application of DACE significantly suppressed the growth of mouse NSCLC that arises from type II alveolar pneumocytes due to constitutive expression of a human oncogenic c-RAF kinase (c-RAF-1-BxB) transgene in these cells. Taken together, these findings suggest that DACE is a promising lead compound for the development of an anti-lung-cancer drug.

## Introduction

Lung cancer is the most deadly type of cancer in humans causing approximately 1.38 million deaths annually worldwide [1]. The most common form is non-small-cell lung cancer (NSCLC), and adenocarcinoma is the most prevalent histology present in 50% of all NSCLCs [2]. There is an unquestionable urge to develop new and effective treatments for the management of this cancer.

One of the well-known hallmarks of cancer is the deregulation of apoptosis (i.e., programmed cell death) [3]. Several promising targets for intervention have been identified by studying the molecular abnormalities that underlie tumorigenesis, such as the signal transduction pathways that regulate apoptosis. One of these targets is the epidermal growth factor receptor (EGFR), which is a member of the ErbB family with signal-transducing tyrosine kinase activity, located in or at the cell membrane [4]. EGFR activation triggers a network of signal transduction cascades that includes activation of PI3K/AKT, RAS/RAF/ERK, and JAK/STAT signaling pathways. These pathways lead to stimulation or inhibition of transcription factors that regulate expression of both pro- and anti-apoptotic genes, effectively disturbing the apoptotic machinery [4,5]. EGFR has been implicated in regulating growth and survival of NSCLC, with overexpression occurring in 45% to 70% of the cases, which is also accompanied by a constitutive activation of the major downstream EGFR effector proteins including PI3K [6], AKT [7], ERK [8], and STAT3 [9].

Natural plant products have been traditionally used for preventing and treating several diseases, including cancer [10]. Moreover, natural products serve as an important source of chemotherapeutic drugs [11,12] and hence approximately 59% of commercially available anti-cancer drugs were directly or indirectly originated from natural sources [13].

In this perspective, cucurbitacins and their derivatives have become a focus of research because of their strong capability to inhibit several types of cancers [14–17]. Cucurbitacins are a group of diverse highly oxygenated triterpenoid molecules predominantly found in different species of the Cucurbitaceae family. They are derived from the cucurbitane skeleton [19-(10→9β)-abeo-10α-lanost-5-ene], which is known for having biological activities including anti-inflammatory, anti-pyretic, analgesic, and hepatoprotective actions [14,18] but the most relevant effects of these molecules are, without doubt, their cytotoxic effects toward a number of human cancer cell lines such as those of the breast [19], lung [20–22], prostate [23,24], and human colon [25,26].

**Fig 1:**
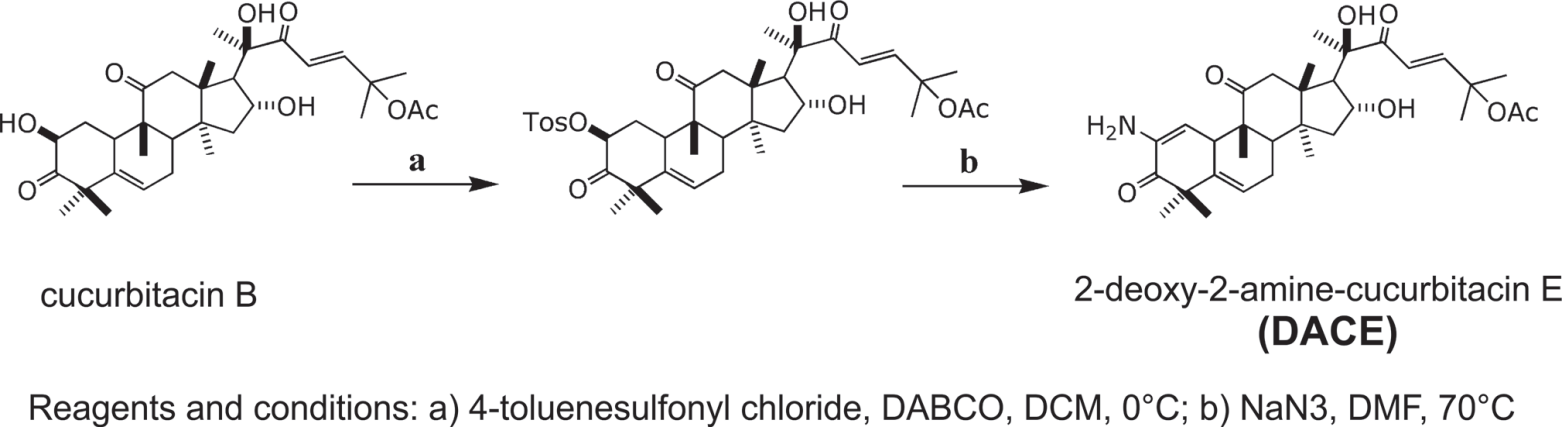
Scheme for preparation of a novel semisynthetic derivative of cucurbitacin B (DACE).

Recently, we described novel cytotoxic cucurbitacins isolated from *Wilbrandia ebracteata* Cogn. [21] and unraveled the apoptotic mechanism in NSCLC cells for the most active compound [27]. We also described new semisynthetic derivatives of cucurbitacin B that are highly cytotoxic against A549 cells [22]. In the present study, we have elucidated the *in vitro* mechanism of cell death induced by a new semisynthetic derivative of cucurbitacin B, the 2-deoxy-2-amine-cucurbitacin E (Fig. 1) (named here as DACE) on A549 cells. We evaluated its effects on cell growth, cell cycle distribution, apoptosis, morphological changes, and expression of regulatory proteins as well as signaling pathways involved in such processes. Furthermore, this potent derivative was also evaluated *in vivo* in a transgenic mouse lung cancer model expressing a mutated and constitutively active c-RAF kinase (c-RAF-1-BxB) under the control of the human surfactant protein C (SP-C) promoter in type II alveolar pneumocytes [28].

## Material and Methods

### Semisynthesis of DACE

The natural precursor cucurbitacin B (200mg, 0.358mmol) was firstly converted into a tosylated intermediate by reaction with *p*-toluenesulfonyl chloride (340mg, 1.432mmol) and DABCO (200mg, 1.79mmol) in dichloromethane (2mL). The next step was the nucleophilic substitution of the intermediate (200mg, 0.28mmol) using NaN_3_ (182mg, 2.8mmol) in DMF (2.5mL), to afford DACE [22] (Fig. 1).

### Cell lines

A549 (human non-small-cell lung cancer), NIH3T3 (immortalized mouse embryo fibroblasts), and NIH3T3 sublines stably retrovirally transduced with RAF or RAS oncogenes [NIH3T3(v-RAF) and NIH3T3(k-RAS), respectively] were described previously [27,29]. They were cultured in Dulbecco's modified Eagle medium (DMEM), supplemented with 10% heat-inactivated fetal bovine serum (FBS), and maintained at 37°C with 5% CO2 and were authenticated by Multiplexion in March 2013. All cell lines were thawed from early-passage frozen stocks and were passaged less than 20 times prior to use. All cells were routinely monitored for microbial and mycoplasma contamination.

### Transient transfections and plasmids

A549 cells were transfected with Lipofectamine 2000 (Life Technologies, USA) according to the manufacturer’s instructions. The plasmid p-ECFP-C1, encoding human EGFR fused to cyan-fluorescent protein, was kindly provided by Dr. N. Zobiack (Institute for Medical Biochemistry, Münster, Germany). Expression constructs for a wild-type form of AKT in pCMV5 were kindly provided by Dr. J. Troppmair (Daniel Swarovski Research Laboratory, University of Innsbruck, Austria), and have been described before [30]. After 24h incubation at 37°C with 5% CO_2_, cells were treated with DACE for 24h and analyzed by Western blotting as described below.

### Cell viability assay

Cell viability was measured using MTT reduction assay [3-(4,5-dimethylthiazol-2-yl)-2,5-diphenyl tetrazolium bromide] (Sigma, MO, USA) [31]. Briefly, cells were seeded in 96-well culture plates (1x10^4^cells/well) and, after 24h, treated with different concentrations of DACE; 48h or 72h later the medium was replaced by 50μL of MTT reagent (1mg/mL) and cells were incubated for a further 4h, followed by reading on a scanning multi-well spectrophotometer (Infinite 1200 TECAN, Grödje, Austria). The 50% inhibition concentration (IC_50_) was defined as the concentration that inhibited cell proliferation by 50% when compared to untreated cells. Paclitaxel (Sigma, MO, USA) (at 0–10μM) was used as a positive control. Final solvent concentration showed no interference with cell growth (data not shown).

### Clonogenic assay

For evaluation of colony-forming capability, the colony formation assay was performed [32]. First, A549 cells were treated for 48h with 0.5 and 1μM of DACE, followed by medium removal. Cells were washed twice with PBS, harvested and reseeded at a density of 200 cells/well in 6-wells plates without treatment. Media was changed every 2 days. After 12 days, colonies were fixed with methanol, followed by staining with 0.1% crystal violet. The plates were photographed, and the number of colonies was counted.

### Cell cycle and apoptosis analysis by flow cytometry

To determine cell cycle progression, A549 cells (5×10^5^) were exposed to DACE in 6-well plates for 24h, washed twice with phosphate-buffered saline (PBS, pH 7.4), centrifuged at 500 X *g* for 5 min, and fixed with 70% ice-cold ethanol at 4°C for 30 min. After fixation, cells were treated with 50μg/ml RNase, and stained with 100μg/ml propidium iodide (PI) for 30 min at room temperature in the dark. Analysis was performed immediately after staining using a FACS Calibur instrument (Becton Dickinson, BD, USA). The percentages of cells in each phase of the cell cycle (G1, S, and G2/M) were determined using the CellQuest Pro software (BD).

Apoptotic populations of vehicle- or 12h DACE-treated cells were quantified using the dual staining Annexin V-FITC/PI apoptosis detection kit (Sigma, MO, USA) according to the manufacturer’s instructions.

### Caspase assay

Caspase-3 protease activity was determined using a commercially available kit (Millipore, MA, USA). Briefly, A549 cells (5x10^5^/six-well) were treated with DACE for 12h. Cells were harvested and resuspended in 150μl of chilled cell-lysis buffer, incubated on ice for 10 min, and centrifuged for 5 min (10,000 X *g*). Then the supernatants were transferred (cytosolic extract) to fresh tubes and put on ice for immediate processing. Cell lysates were tested for protease activity by the addition of a labeled caspase substrate (DEVD-pNA), and incubated at 37°C for 2h. pNA absorbance was quantified using a spectrophotometer (Infinite 1200 TECAN, Grödje, Austria) at a wavelength of 405 nm. Fold-increase in caspase-3 activity was determined as a relation of OD values from experimental to that of control samples.

### Confocal laser scanning microscopy to visualize filamentous actin and nuclei

A549 cells grown on coverslips placed into six-well plates were treated with vehicle or DACE for 12h and then fixed in 4% (w/v) paraformaldehyde at 37°C. Following permeabilization in 0.5% (v/v) Triton X-100, the cells were incubated with TRITC-labeled-phalloidin (Invitrogen, Carlsbad, USA) for F-actin staining, and with Hoechst (Invitrogen, Carlsbad, USA) to detect nuclei. The coverslips were mounted with 80% glycerol in PBS and confocal images were collected on a Leica DMI6000 B microscope (Leica Microsystems, Wetzlar, Germany).

### Western blotting analysis

After treatment, cells were lysed on ice with RIPA lysis buffer (25mM Tris-HCl pH 8.0, 137mM NaCl, 10% glycerol, 0.1% SDS, 0.5% DOC, 1% NP40, 2mM EDTA pH 8.0, 5mg/mL leupeptin, 5mg/mL aprotinin, 0.2mM pefablock, 1mM sodium vanadate, and 5mM benzamidine) for 30 min. Cell lysates were cleared by centrifugation and equal amounts of total protein lysates were separated by sodium dodecyl sulfate polyacrylamide gel electrophoresis (SDS-PAGE) and blotted on nitrocellulose membranes (Schleicher & Schuell, Dassel, Germany). PI3K activity was monitored by detection of PI3K, PTEN, PDK1, and AKT phosphorylation. The antibodies used were against: PI3Kinase p85 [Tyr458]/p55 [Tyr199], PTEN [Ser380/Thr382/383], PDK1 [Ser241] purchased from Cell Signaling Technology, Danvers, USA (CST), and AKT [Ser473] from Invitrogen. Phosphorylated forms of EGFR, ERK, and STAT3 were detected with phosphospecific antibodies against ERK1/2 [Thr202/Tyr204] and EGFR [Tyr1068] from CST and STAT3 [Tyr705] from Millipore. Endogenous and over-expressed EGFR or AKT were detected by an EGFR- or AKT-specific rabbit antiserum, respectively (CST). Other antibodies used were specific for: cleaved caspase-3, phospho-p38 MAPK [Thr180/Tyr182], phospho-NFκB p65 [Ser536], cyclin B1, survivin, and NFκB p65 (all purchased from CST). The anti-phospho-JNK/SAPK [Thr183/Tyr185] antibody was from BD, and the anti-p38α, IκBα, and JNK 1/3 antibodies were from Santa Cruz Biotechnology (SCB, Dallas, USA). In some of the assays, loading controls were performed with anti-ERK2 (C-14, SCB), anti-AKT (CST), anti-β-actin (Millipore), or anti-α-tubulin antibodies (Sigma). After incubation with the corresponding secondary antibodies conjugated to horseradish peroxidase, protein bands were revealed by Pierce ECL substrate (Thermo Scientific, MA, USA), according to the manufacturer’s protocol. The relative expression of the c-RAF-1-BxB oncogene protein in lung tissues was quantified after resolving RIPA tissue lysates by SDS-PAGE and using the Advanced Image Data Analyzer Software (AIDA, Raytest, GmbH, Straubenhardt, Germany). The total band densities were measured against the local background and normalized to the density of the appropriate beta-actin loading control bands. The values of untreated control samples were taken as unity.

### Animals

All animals were cared for and handled in strict accordance with good animal practices as defined by the EU Council Directive 2010/63/EU, the German Animal Welfare Act “TierSchG” and to the institutional guidelines of the Westfaelian-Wilhelms University Münster and have been approved by the responsible authority (Landesamt für Natur, Umwelt und Verbraucherschutz Nordrhein-Westfalen (LANUV-NRW)), Germany.

The anti-tumor activity of DACE was evaluated in a transgenic mouse model of c-RAF-induced lung adenoma (c-RAF-1-BxB mice). The mice contain a lung-targeted expression of the NH2-terminal deleted human c-RAF-1-BxB oncogenic mutant. The oncogene is controlled by the human SP-C promoter, allowing selective expression in the type II epithelial cells that line the lung alveoli. Consequently, in the lungs of c-RAF-1-BxB mice, single tumor foci are detectable in the alveolar area by the age of 8 weeks, which develop to solid tumors [28]. Broods of 4 months old homozygous transgenic mice (male) were randomly assigned to the control group (*n* = 4) and to the study group (*n* = 4). DACE was administered daily by intraperitoneal injections at a dose of 1mg/kg for 21 days. Then, mice were sacrificed under deep anesthesia with isoflurane, lungs were isolated and analyzed by immunohistochemistry, quantitative real-time PCR, and Western blotting for the extent of tumor tissue, c-RAF-1-BxB mRNA, and protein expression, respectively.

### Histology and immunohistochemistry

For lung analysis, animals were euthanized and the right lungs fixed with 4% paraformaldehyde for histological studies. The left lungs were minced and one half was homogenized in RNAlater solution for qRT-PCR studies and the other half in RIPA buffer for protein analysis. Paraffin sections (4μm) of lung specimens were de-paraffinized and stained according to standard protocols with hematoxylin and eosin. For immunohistochemistry, paraffin sections were dewaxed, rehydrated, and boiled in 10mM sodium citrate buffer (pH 6.0) for antigen retrieval, blocked with 5% FBS, and incubated with rabbit-anti-human-RAF antibodies [28] for 1h at room temperature. The SignalStain Boost IHC Detection Reagent (HRP, Rabbit) from Cell Signaling Technology, Danvers, USA was used to detect the stained protein. Counterstaining with hematoxylin allowed nuclei visualization. Usually, 4 different sections per mouse sample and 4 mice per experimental setting were quantified. Tumor foci were qualified as RAF-positive foci containing at least 10 nucleated cells. The area of the RAF-positive tumor foci were then measured and taken in relation to the total section area of the specimen. Usually, 4 sections per mouse sample representing different lung parts were quantified in a blinded fashion. All analyses were performed in a blinded fashion.

### mRNA isolation, cDNA synthesis, and qRT-PCR

Total lung RNA was isolated using Trizol reagent (Invitrogen) according to the manufacturer’s instructions. cDNA was synthesized from 1μg of total RNA using RevertAid H Minus M-MuLV Reverse Transcriptase (Fermentas, St. Leon-Rot, Germany) according to the protocol of the manufacturer. The mRNA levels were determined by TaqMan qRT-PCR using the LightCycler 480II (Roche Diagnostics, Mannheim, Germany). Each cDNA probe was analyzed in triplicate and specific signals were scored in relation to the signals of two housekeeping gene transcripts, cytochrome *c* and GAPDH. The results from different experiments were normalized to the expression of a calibrator probe, which was applied as a positive control in each experiment. Primers for an intronic region of the IL-2 gene were always included to ascertain that the probes were not contaminated with genomic DNA. The primers used were assigned using the Universal ProbeLibrary Assay Design Centre at www.roche-applied-science.com/sis and were: 5’-GCTACCCATGGTCTCATCGT-3’ and 5’-GAAACCCCTCCGAATGCT-3’ for cytochrome *c*; 5’-TCACATCCAGTTCTATGCTGGT-3’ and 5’-CAAGGAAACTGGGAACATGAA-3’ for IL-2; 5’-GAGGATATGCCTCCCCAGA-3’ and 5’-TTTCTTCACACAGTCAGCTACCA-3’ for c-RAF-1-BxB; 5’-TGTCCGTCGTGGATCTGAC-3’ and 5’-CCTGCTTCACCACCTTCTTG-3’for GAPDH. The changes in gene transcription were ascertained as differences between the transcription of the housekeeping genes and the gene of interest using the 2^-ΔΔCT^ method [33].

### Statistical analysis

GraphPad Prism 5 Software (San Diego, CA) was used to calculate IC_50_ values and their 95% confidence intervals (CI) through a nonlinear fit-curve (log of compound concentration *versus* normalized response–variable slope). Statistical analysis was performed by one-way analysis of variance (ANOVA) followed by the Dunnett test, with P values <0.05 regarded as statistically significant and *P<0.05, **P<0.01, ***P<0.001.

## Results

### DACE inhibits the growth of A549 cells in vitro and reduces their colony-forming capability

**Fig 2:**
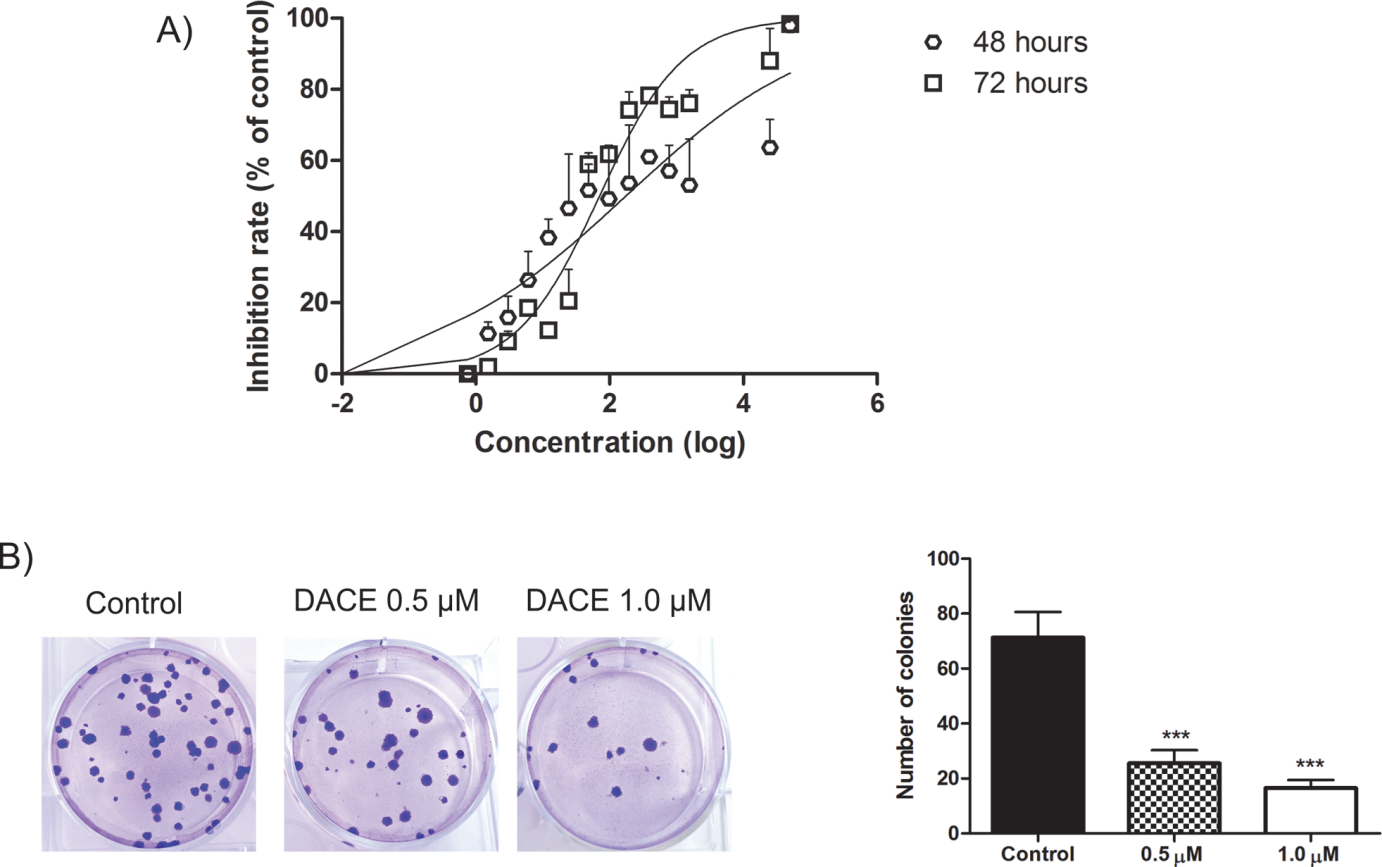
Cell and clonogenic growth inhibition by DACE. (A) Human non-small cell lung cancer cells (A549 cells) were treated with different concentrations of the DACE for 48h and 72h. The growth inhibition effects were determined by MTT assay and the IC_50_ was calculated by GraphPad Prism 5 software through a nonlinear fit-curve (log of compound concentration *versus* normalized response—variable slope). (B) A549 cells were treated with 0.5 and 1 μM of the DACE for 48h, followed by two washes for compound removal. Then, cells were plated for clonogenic assay; left—representative images of colonies formed from A549 cells under the different treatment conditions; right—number of colonies after 12 days. ***p<0.001 as compared with control.

Initially, we explored whether DACE affects the proliferation of A549 human lung adenocarcinoma cells using MTT assay. Results of Fig. 2A show that DACE potently inhibited cellular proliferation in a concentration- and time-dependent manner. The calculated IC_50_ values for 48h and 72h were 0.42μM and 0.12μM, respectively (equivalent to log value of 2.62 and 2.075). To test the long term effect of DACE treatment, A549 cells were treated for 48h and then replated with low density into new plates without further treatment. Quantification of colonies formed twelve days after DACE treatment showed that their number was reduced compared to control cells to 64% and 76%, respectively, in both tested concentrations (*p*<0.001 vs. control) (Fig. 2B).

### DACE leads to cell cycle arrest and increased apoptosis in A549 cells

**Fig 3:**
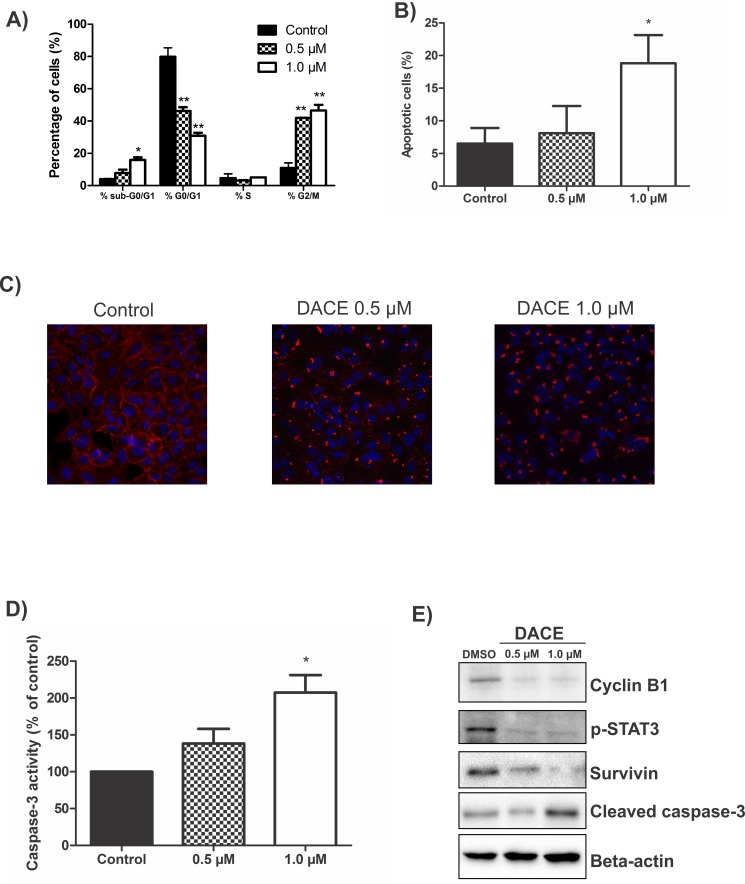
Effects of DACE on cell cycle arrest and apoptosis. (A) A549 cells (5x10^5^) were treated with DACE and analyzed 24h later by flow cytometry. The values indicate the percentage of A549 cells in the indicated phases of the cell cycle (sub-G0/G1, G0/G1, S and G2/M). *p<0.001 and **p<0.0001 as compared with control. (B) The A549 cells were treated for 12h with DACE, stained with Annexin V/PI, and submitted to flow cytometry for analysis of the apoptotic cell proportion. *p<0.05 as compared with control. (C) The A549 cells were either untreated or treated with 0.5μM and 1.0μM DACE for 12h, fixed, stained with Hoechst and TRITC-labeled-phalloidin and analyzed by confocal microscopy. Overlay images are shown. (D) The A549 cells were treated for 12h with DACE and their cytosolic fraction was analyzed for changes in the activity of caspase-3. *p<0.05 as compared with control. (E) A549 cells were treated with DACE for 12h and then subjected to Western blotting using antibodies as indicated. Equal protein loading was confirmed by probing for beta-actin. Representative images of three independently repeated experiments are shown. The values represent means of three independent experiments and SD.

To better understand the mechanism by which cell proliferation was suppressed by DACE, we investigated the distribution of cell cycle phases of A549 cells following treatment with DACE by FACS analysis. The untreated control cells showed a typical distribution of G0/G1, G2/M, and S phases, but 24h exposure of cells to DACE caused a significant enrichment of cells in the G2/M phase in a concentration-dependent manner (Fig. 3A). An increase from 10.97±3.04% to 41.92±0.20% and 46.56±6.01% cells in G2/M phase was detected after the treatment with 0.5μM and 1μM of DACE, respectively (*p*<0.0001 vs. control). This was followed by a reduction in G0/G1-phase cells: from 79.75±5.58% in the controls to 46.23±4.03% and 30.88±3.18% in samples treated with 0.5μM and 1μM of DACE, respectively) (*p*<0.0001 vs. control). These results indicated that the compound inhibits the growth of A549 cells by arresting them in the G2/M cell cycle phase.

Interestingly, the FACS analysis of cell cycle distribution with PI staining showed that the treatment of A549 cells with DACE results in a significant increase of the sub-G0/G1 peak, suggesting that DACE might induce cell apoptosis. The percentage of G0/G1 cells was increased from 4.02±0.33% in the control group to 15.90±2.80% after 24h of treatment with 1μM of DACE (*p*<0.001 vs. control). To investigate if the observed sub-G0/G1 peak was due to induction of apoptosis, the cells were double stained with PI (which intercalates in the DNA of necrotic and late apoptotic cells) and Annexin V conjugated–FITC (which only binds early apoptotic cells). A 12h exposure of cells to 1.0μM of DACE indeed increased the percentage of apoptotic cells from 6.50±2.41% in the untreated controls to 18.83±4.30% in treated cells (*p*<0.05 vs. control; Fig. 3B). Thus, the treatment of A549 cells with DACE not only arrested cell division but also triggered apoptosis.

### DACE induces intense cytoskeleton changes and caspase-3 activation

A prominent feature of DACE was the induction of rapid morphological alterations of A549 cells. Appearance of plasma membrane protrusions, so-called blebs, was observed within minutes of DACE treatment, which is a typical feature of apoptosis (data not shown). Since a correlation between surface bleb formation and perturbation of normal actin cytoskeletal organization has been reported [34], we analyzed whether DACE affects the F-actin microfilament structures. As shown in Fig. 3C, the untreated cells displayed a regularly organized actin cytoskeleton that was, as is typical for epithelial cells, prominently expressed at cell-cell contacts. However, 12h exposure to 0.5μM or 1.0μM of DACE triggered a collapse of microfilaments, resulting in the formation of irregular and globular aggregates of F-actin in the cytoplasm near to the nuclei (Fig. 3C). This observation further emphasizes the pro-apoptotic potential of the DACE. It is well known that caspases are executors of the apoptotic process and are activated by proteolytic cleavage of pro-caspase forms [35]. To evaluate the effects of DACE on the activation of caspase-3, the cytosolic extracts of treated or untreated control cells were incubated with a caspase-3-specific substrate. As shown in Fig. 3D, 12h treatment of A549 cells with 1μM of DACE caused a significant increase in the proteolytic activity of caspase-3. Thus, the morphological signs of apoptosis induction correlate well with caspase-3 activation.

### DACE suppresses STAT3 activation and apoptotic-related proteins

In order to understand the mechanism involved in the DACE action on cell cycle and apoptosis, we investigated its effect on the levels of cyclin-B1, phospho-STAT3, survivin, and cleaved-caspase 3 by Western blotting. In accordance with results of cell proliferation analyses, the expression of cyclin-B1, an important cell cycle regulator [36], was reduced, especially after exposure to 1.0μM of DACE (Fig. 3E). Also, phosphorylation of the latent cytosolic transcription factor STAT3 that is aberrantly activated in many cancers, including NSCLC and is responsible for up-regulated expression of several cell cycle regulators and anti-apoptotic proteins, was significantly decreased by DACE. In addition, cell treatment with DACE led to reduced expression of survivin, a well-known anti-apoptotic protein [37], but to increased cleavage of caspase 3. These data are in good agreement with the ability of DACE to activate caspase-3, demonstrated by the cleavage of a caspase-3-specific substrate (Fig. 3D). Taken together, these results showed that DACE induced cell cycle arrest and apoptosis by affecting key kinases and other enzymes that regulate these physiological processes.

### DACE is a potent suppressor of the PI3Kinase/AKT signaling pathway and alters the phosphorylation/activation status of ERK

**Fig 4:**
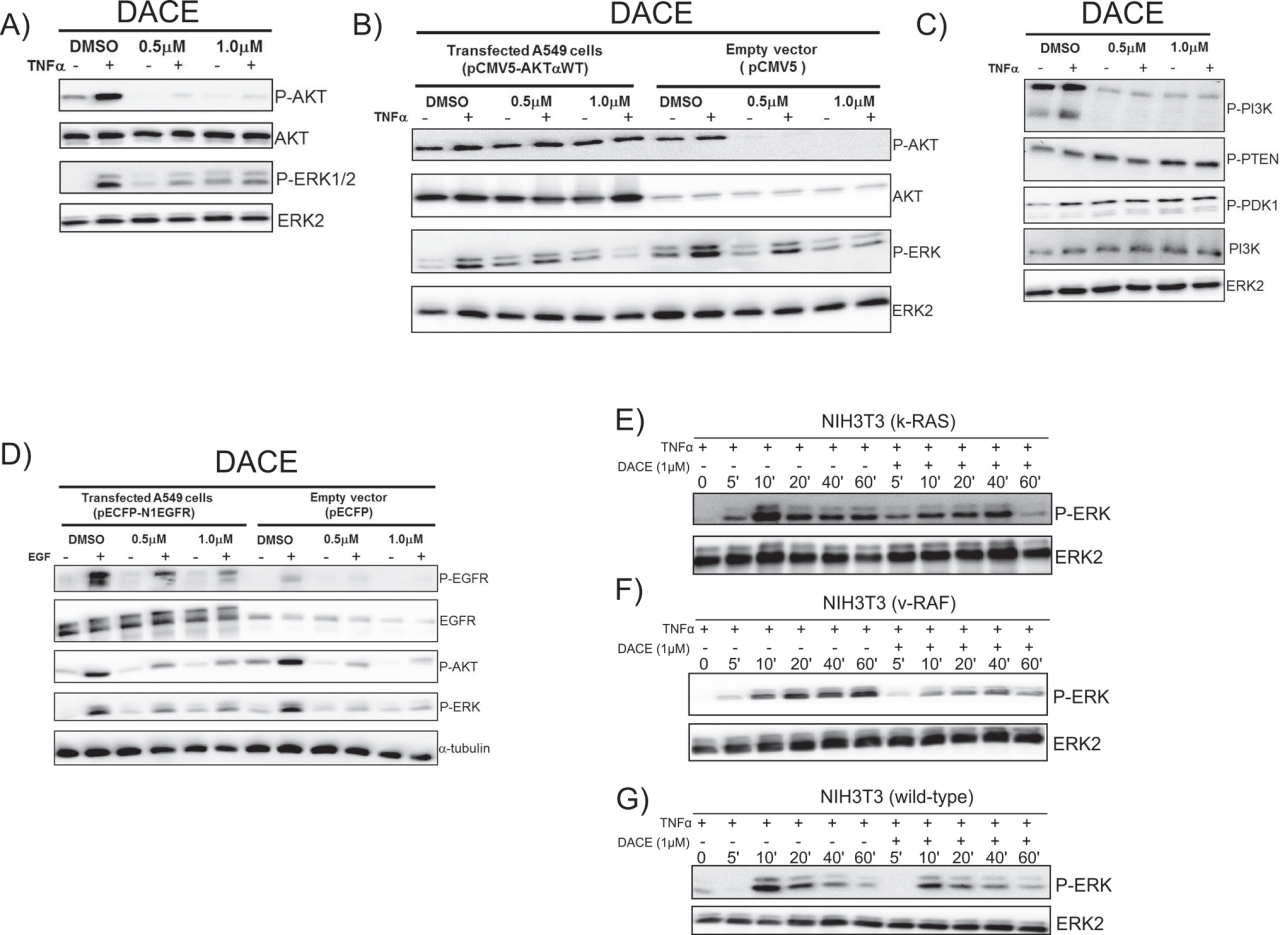
Effects of DACE on TNFα-mediated activation of signaling pathways. (A) Effect of DACE on the phosphorylation status of AKT and ERK. (B) Effect of DACE on the phosphorylation status of AKT and ERK in A549 cells transiently transfected with 1μg of wild-type form of AKT or the empty pCMV5 vector. (C) Effect of DACE on the phosphorylation status of PI3K and its regulators PTEN and PDK1. In A, B, and C, the cells were exposed to 0.5μM and 1.0μM of DACE for 24h, stimulated or not for an additional 15 min with 30ng/mL TNFα and analyzed by Western blotting. (D) Effect of DACE on the phosphorylation level of EGFR, measured by phosphorylation of its specific Tyr 1068 site and downstream targets AKT and ERK. The A549 cells were transiently transfected with 1μg human EGFR or its comparable empty-vector control. The cells were exposed to 0.5μM and 1.0μM of DACE for 24h, stimulated or not with EGF (10ng/mL, 15min) and then analyzed by Western blotting. (E, F and G) Effect of DACE on the phosphorylation status of ERK in NIH3T3(k-RAS)- (E), NIH3T3(v-RAF)- transformed cells (F), and NIH3T3(wild-type) cells (G). The cells were simultaneously stimulated with TNFα (30ng/mL) and exposed or not to 1.0μM DACE for timepoints indicated and analyzed by Western blotting. Equal protein loading was confirmed by probing for tubulin or ERK2. The most representative results of three independent experiments are shown.

To detect signaling pathways that might be affected by DACE, human non-small-cell lung cancer A549 cells were treated with DACE for 24h followed by stimulation with TNFα (30ng/mL) for an additional 15 min. TNF activates a broad spectrum of different signaling cascades, thus allowing narrowing down the affected pathways in the same experimental setting. As PI3K/AKT and RAS/RAF/ERK pathways are key regulators of cell survival and proliferation, and their deregulation is commonly found in tumors [4], special attention was paid to these cascades. As shown in Fig. 4A, the TNFα-mediated activation/phosphorylation of both AKT and ERK was inhibited when A549 cells were treated with either 0.5μM or 1.0μM of the compound. In contrast, the activation state of key signaling proteins after TNF stimulation, such as p38, JNK, and NF-kappaB p65 as well as the degradation of IκBα, was not significantly changed upon treatment with DACE (data not shown). To reveal whether DACE affects the AKT kinase directly, A549 cells were transiently transfected with either empty vector or a wild-type form of AKT and exposed to 0.5μM and 1.0μM of DACE for 24h. The expression of recombinant AKT kinase caused increased AKT phosphorylation in non-stimulated cells and, interestingly, this basal phosphorylation was not inhibited by DACE. In contrast, phosphorylation of endogenous AKT was completely inhibited by DACE (Fig. 4B). Overexpression of AKT also had no effect on inhibition of ERK phosphorylation by DACE. These data indicate that upstream components of the AKT signaling cascade, rather than AKT itself, are affected by DACE. However, while phosphorylation of AKT and PI3K were strongly inhibited by both 0.5μM and 1.0μM of DACE, the phosphorylation of upstream components of PI3K/AKT signaling pathway such as PTEN and PDK1 remained unaffected (Fig. 4C).

### DACE inhibits the expression level of EGFR and phosphorylation of EGFR

Both PI3K and ERK1/2 kinases can be activated by tyrosine kinase receptors [4]. EGFR is expressed at high levels in a wide range of tumor types and in most lung cancers it has been associated with lower rates of survival. Hence, we assessed whether the observed effects of DACE on PI3K/AKT and ERK1/2 might result from inhibition of EGFR. Human EGFR was overexpressed in A549 cells and its phosphorylation was analyzed after stimulation with EGF in the presence or absence of DACE. Growth factor stimulation resulted in a stronger tyrosine phosphorylation signal of the overexpressed receptor than in empty-vector transfected cells, while incubation with DACE efficiently inhibited phosphorylation in both vector- and EGFR-transfected cells. The effect was concentration-dependent and a concentration of 1μM of DACE was sufficient to completely prevent the phosphorylation of endogenous EGFR and to efficiently inhibit phosphorylation in overexpressing cells (Fig. 4D). Interestingly, overexpression of EGFR also attenuated the inhibitory effect of DACE on AKT and ERK kinases, suggesting that the tyrosine kinase receptor is probably the primary target of the compound.

### DACE shows selectivity towards malignant transformed cells

In NSCLC, the RAS-RAF-MEK-ERK signaling pathway is frequently hyperactivated [5]. Our experiments showed that DACE efficiently inhibited the activation of this pathway. To investigate the selectivity and efficiency of DACE towards oncogenic activation of the RAS-RAF-MEK-ERK pathway, we analyzed established NIH3T3 model cell lines, the oncogenic transformation of which is based on constitutive oncogenic activation of either RAS or RAF proteins [28,38,39]. The growth inhibitory effect of DACE towards v-RAF and k-RAS transformed cells was 31- or 5-times more efficient, respectively, than towards wild-type non-transformed NIH3T3 cells. For comparison, staurosporine, which is an inhibitor of diverse signaling kinases had either no effect or even a growth promoting effect compared to wild-type cells (Table 1). In good agreement with these data, the TNFα-mediated phosphorylation of ERK1/2 was efficiently blocked by DACE in these cells and the inhibitory effect was more pronounced in v-RAF-compared with k-RAS-transformed NIH3T3 fibroblasts (Fig. 4E-4F).

**Table 1:** Growth inhibitory effects and selectivity of DACE and staurosporine towards wild-type and v-RAF- and k-RAS-transformed NIH3T3 fibroblasts.

Cells	DACE	Staurosporine
	IC_50_ ^a^ (CI 95%)	IC_50_ ^a^ (CI 95%)
NIH3T3(wild-type)	17.592 (9.872–31.349)	0.0692 (0.029–0.165)
NIH3T3(v-RAF)	0.561 (0.316–0.998)	0.064 (0.048–0.088)
NIH3T3(k-RAS)	3.559 (1.612–7.857)	12.728 (0.748–21.643)
SI^b^ NIH3T3(v-RAF)	31.4	1.08
SI^b^ NIH3T3(k-RAS)	4	ND

ND = Not determined, since staurosporine was more cytotoxic toward normal NIH3T3 cell line.

^a^IC_50_ (μM): inhibitory concentration of 50% cell growth was calculated through a nonlinear fit-curve (log of compound concentration *versus* normalized response—variable slope)

^b^Selectivity index: calculated as IC_50_ NIH3T3(wild-type)/IC_50_ NIH3T3(v-RAF) or IC_50_ NIH3T3(wild-type)/IC_50_ NIH3T3(k-RAS).

### DACE reduces RAF-induced lung tumors growth *in vivo*

**Fig 5:**
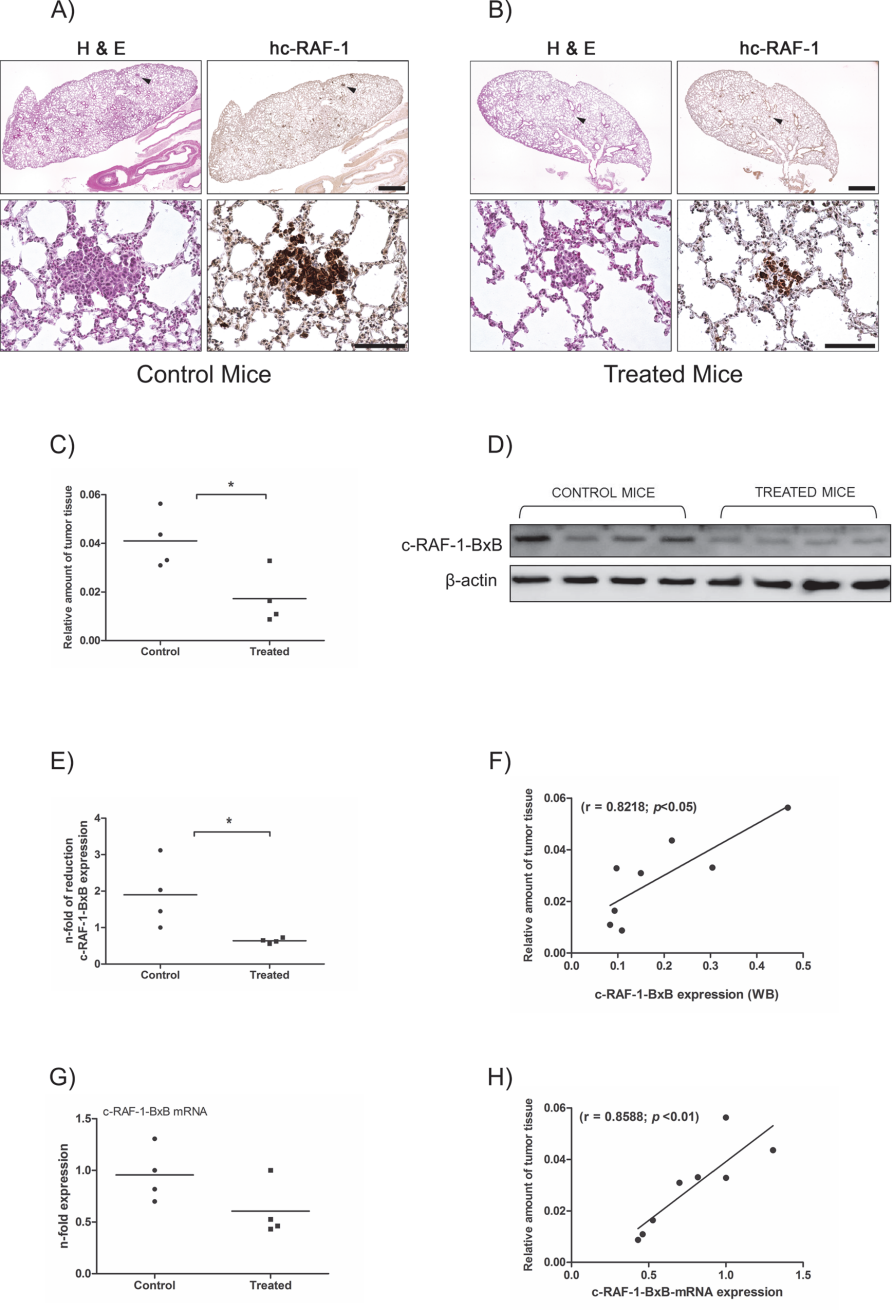
Effects of DACE on c-RAF-1-induced lung tumor growth in mice. (A and B) c-RAF-1-BxB transgenic mice were injected daily with either DMSO (n = 4, A) or 1 mg/kg of DACE (n = 4, B). On day 21, the lungs were fixed, embedded into paraffin and stained for H&E (left images) or for human c-RAF-1 protein (right images). Bars, 1000 μm for upper images and 100 μm for lower images. Arrow heads on upper images indicate lung tumor areas shown on lower images. (C) The total amount of tumor tissue in the lungs of untreated control mice (*n =* 4) and DACE-treated mice (*n =* 4) measured after immunohistochemistry. The lungs of treated mice exhibited 58% (*p<0.05, *t* test) less tumor tissue in comparison to untreated control animals. (D) Western blotting of tumor lysates of untreated control mice (*n* = 4) and DACE-treated mice (*n* = 4) for c-RAF-1-BxB expression. Beta-actin was used as a loading control. (E) Densitometric quantitation of the human c-RAF-1-BxB protein expressed in the lungs of untreated and DACE-treated mice. The lungs of treated mice exhibited 66% (*p<0.05, *t* test) less c-RAF-1-BxB expressed protein in comparison to untreated control animals. (G) Total RNA was isolated from the lungs of untreated mice (*n =* 4) and DACE-treated mice (*n* = 4), reverse transcribed, and the expression of c-RAF-1-BxB mRNA was determined by quantitative real-time PCR. The expression of c-RAF-1-BxB mRNA was reduced by 37% after systemic treatment with DACE, albeit the means are not statistically significant when compared by *t* test. (*p*>0.05). Relationship between tumor tissue amount and c-RAF-1-BxB protein (F) and c-RAF-1-BxB mRNA (H) in lungs of all mice analyzed. The relative amounts of tumors, and c-RAF-1-BxB protein and mRNA, were measured by immunohistochemistry, Western blotting, and qRT-PCR as shown in (A/B, D and G).

To study the *in vivo* relevance of DACE in preventing tumor growth, we injected the compound into the peritoneal cavity of c-RAF-1-BxB mice. These transgenic mice develop multiple adenomas of type II pneumocyte origin within 4 months after birth. Similar to NIH3T3(v-RAF) cells, these mice contain a truncated oncogenic version of the human RAF kinase, the expression of which, however, is restricted to type II lung epithelial cells, as the c-RAF-1-BxB transgene is driven by the surfactant protein-C (SP-C) promoter [28]. Thus, the development of NSCLC–like tumors by these mice is driven mainly by the oncogenic human RAF kinase. Histological analyses of H&E stained lung tissue of 4-month-old mice that have been treated with 1mg/kg of DACE every second day for 21 days revealed a slight decrease in the number and size of tumor foci (16% tumor reduction was measured, data not shown). In addition, immunohistochemical study of lung sections with an antibody recognizing the human c-RAF-1 kinase confirmed that tumor but not normal lung tissue was affected and that the DMSO control did not show any effect (Fig. 5A). At this dose and scheme of treatment, DACE was well tolerated (data not shown). However, when DACE was administrated at the same dose (1mg/kg) and for the same time period (21 days), but every single day, which was still tolerated by the animals, the size of c-RAF-BxB-1-positive tumor foci was significantly reduced compared to the control-treated mice (Fig. 5A-C). As the development of lung adenomas in these mice mainly depends on expression of the oncogenic human c-RAF-1-BxB [28], it is conceivable that the amount of c-RAF-1-BxB protein or mRNA in lungs will be directly proportional to the amount of tumor tissue. Indeed, not only the number of tumor colonies and their size were reduced after treatment of mice with DACE, but also the amount of c-RAF-BxB protein and mRNA (Fig. 5D-G) and the measured amounts of tumor tissue correlated very well with the amounts of expressed human c-RAF-1-BxB protein and mRNA (Fig. 5F-5H).

## Discussion

Cucurbitacins are a class of highly oxidized tetracyclic triterpenoids that exhibit a wide array of *in vitro* and *in vivo* pharmacological effects, including anti-tumor activity [14–17]. In our previous studies, we described a novel semisynthetic derivative of cucurbitacin B (DACE) as a potential anti-cancer agent. DACE showed a similar high cytotoxic activity against A549 cells as its precursor cucurbitacin B [22]. In the plant kingdom there are no natural cucurbitacins that have an amino group in C-2, and this makes DACE unique in its class. Considering the simple and short synthetic route starting from cucurbitacin B, this compound could be considered as a potential drug leader for the generation of new families of bioactive compounds from natural renewable resources. In this molecule, there are two important features to consider: the double bond between C1 and C2 with the polar group attached in position 2 (in this case nitrogen), and the Michael acceptor in the side chain. These functionalities have been validated as pharmacophores in our previous QSAR work [40]. Changing the hydroxyl group by the amino moiety in C2, DACE has a nucleophilic, basic and hydrogen bond donor/acceptor group instead of a donor/acceptor group. Furthermore, the C-2 altered its hybridization from sp3 to sp2 showing conformational and structural changes in ring A and the whole molecule. These modifications could have an effect in the mechanism of action, solubility and bioavailability of this compound and its derivatives compared with the parental natural compounds.

In this study, we demonstrate for the first time that the semisynthetic derivative of cucurbitacin B (DACE) is a potent suppressor of human NSCLC cell growth *in vitro* through its effects on the actin-cytoskeleton, EGF receptor and its downstream effectors PI3Kinase, ERK1/2 and STAT3, and apoptotic proteins. In addition, for the first time, the potential anti-tumor activity of DACE was shown in a well-established c-RAF-1-induced lung adenoma model *in vivo*.

Cell cycle analysis suggests that DACE inhibits cell growth via blocking cell division at the G2/M phase. In addition, treatment of A549 cells resulted in increased apoptosis (Fig. 3B). These results are in good agreement with recent findings demonstrating that other cucurbitacins also induce a G2/M arrest and apoptosis in other human cancer cell lines *in vitro* [25,41,42]. To better understand the mechanism of DACE-induced cell cycle arrest and apoptosis, we firstly investigated the status of key proteins known to regulate G2/M transition and cell death. The signal transducer and activator of transcription protein 3 (STAT3) is a latent cytosolic transcription factor that is aberrantly activated in many cancers, including NSCLC. In addition, STAT3 is responsible for up-regulating the expression of several cell cycle regulators and anti-apoptotic proteins [43]. Some studies have proposed that cucurbitacins induce inhibitory effects against several human cancer cell lines via suppression of STAT3 phosphorylation [44,45]. In the current study, it was clearly demonstrated that the levels of p-STAT3 and cyclin B1 proteins were decreased in cells treated with DACE (Fig. 3E), suggesting that cell cycle arrest and apoptosis was at least partially mediated via suppression of cyclin B1 and STAT3 activation. The transition from one cell cycle phase to another occurs in an orderly fashion and is regulated by different cellular proteins, such as cyclin B1, which is necessary for the progression of the cells into and out of M phase of the cell cycle. For several drugs, the anticancer effects are mediated by cell cycle arrest and involve modulation of the cyclins and cyclin-dependent kinases that regulate cell cycle progression [46]. In the same context, survivin, which is an Inhibitor of Apoptosis Protein (IAP) that plays an important role in both cell cycle regulation and inhibition of apoptosis, is expressed in cells during the G2/M phase of the cell cycle, followed by a rapid decay of both mRNA and protein levels at the G1 phase [47]. Inhibition of survivin expression or disruption of survivin interaction with microtubules leads to apoptosis at the G2/M phase [48]. Survivin seems to inhibit apoptosis via interactions with multiple regulators of both intrinsic and extrinsic apoptosis pathways [49]. Some published reports suggest that survivin could directly suppress the activity of the effector caspase-3 [50], whereas others propose that survivin primarily acts at the level of an initiator caspase by binding to caspase-9 and/or neutralizing proapoptotic proteins [51]. Therefore, the decreases in survivin levels have been found to result in apoptosis [52]. As this protein is also regulated by STAT3 [53], the decrease in its level by DACE treatment might be because of the inhibition of STAT3. As can be seen in Fig. 3E, both analyzed concentrations of DACE decreased the expression of survivin, indicating that survivin might be a regulator of cell division and be required to protect cells from apoptosis.

The apoptotic nature of cell death in DACE-treated cells was verified by the ability to activate the major effector caspase, caspase-3 [35] (Fig. 3D-3E). This confirms the involvement of this enzyme in the DACE-mediated cell death. Furthermore, DACE induced intense alterations of the cytoskeleton (Fig. 3C). The actin filamentous organization was rapidly disrupted switching from a well-developed network that elongated continuously through the cytoplasm in untreated cells to an intense accumulation of the F-actin next to the cell nucleus in treated cells. These findings are in agreement with the present understanding about cucurbitacins, which suggest a direct modulation of the actin cytoskeleton [23].

Cucurbitacins have shown strong anti-proliferative activity against many human cancer cells, primarily as inhibitors of the JAK/STAT3 pathway, as previously described in this section. Blaskovich and co-workers [54] were the first to report that cucurbitacin I rapidly suppresses phosphorylation of STAT3 in v-Src-transformed NIH3T3 cells and in A549 cells. Consequently, STAT3 DNA binding and STAT3-mediated gene transcription is inhibited. This cucurbitacin also decreased the phosphorylation of JAK without affecting AKT, ERK1/2, or JNK, suggesting that cucurbitacin I is highly selective for JAK/STAT3. Conversely, cucurbitacin Q selectively inhibits STAT3 without affecting JAK2, Src, AKT, ERK, or JNK in A549 cells [44]. Nonetheless, five different leukemia cell lines treated with cucurbitacin B showed cell growth inhibition, which was achieved by suppression of both STAT3 activation and RAF/ERK kinase pathways [55]. However, when hepatocellular carcinoma (BEL-7402) cells were treated with cucurbitacin B, c-RAF was inhibited without affecting STAT3 [56]. Another study showed that cucurbitacin B inhibited the proliferation of seven human osteosarcoma cells lines *in vitro*, suppressing ERK, AKT, and mammalian target of rapamycin (mTOR) proteins [57]. Guo and colleagues[58] showed, for the first time, that cucurbitacin B induced DNA damage in A549 cells mediated by increasing intracellular Reactive Oxygen Species (ROS) formation, which in turn activated ATM-Chk1-Cdc25C-Cdk1 pathway joined by ATM-p53–14–3–3-σ branch, leading to the G2/M phase arrest. The same authors [59], demonstrated the DNA damage induced by cucurbitacin B through ROS increase formation, as well as G2/M arrest and apoptosis in leukemia K562 cells. Another novel target of cucurbitacin B in breast cancer *in vitro* and *in vivo*, which was raised by Gupta and Srivastava [60], is the inhibition of HER2-Integrin signaling. Therefore, cucurbitacins can selectively inhibit different signaling pathways, depending on the cancer cell type, which led us to study which other pathways are affected by DACE.

Here, we demonstrated that DACE can target directly the epidermal growth factor receptor (EGFR) (Fig. 4D), suppressing its phosphorylation in a concentration-dependent manner in both EGFR-overexpressing A549 cells and empty vector control cells, followed by inhibition of its secondary PI3K/AKT, RAS/ERK, and JAK/STAT3 signaling pathways (Fig. 3E and Fig. 4). Genetic analysis of NSCLC revealed that the EGFR and its downstream targets, the members of the mitogenic RAS/RAF/MEK/ERK signalling cascade, are frequently mutated or overexpressed in NSCLC types of tumours. The extent of ERK1/2 activation for example correlated with aggressiveness of NSCLC tumours and was associated with poor prognosis in patients [61,62] and constitutive EGFR-mediated signaling is usually associated with poor prognosis of many human malignancies [4,5]. Emerging evidence indicates that the RAF/MEK/ERK pathway is intimately linked with the PI3K/AKT pathway [63]. In fact, RAS regulates activation of both kinase pathways resulting in the phosphorylation of many downstream targets. ERK activation leads to the induction of genes, such as Bcl-2, caspase-9, and cyclin D1, regulating growth and survival [64]. Likewise, activated AKT exerts its anti-apoptotic activity by preventing the release of cytochrome *c*, inhibiting G2/M checkpoint initiation, and directly inactivating pro-apoptotic factors such as Bad and procaspase-9 [7,65]. Thus, considerable attention has been given to targeting the EGFR pathway for anti-cancer therapy.

To explore whether DACE also have an anti-tumor activity *in vivo*, we decided to target non-small-cell lung cancer (NSCLC) cells. The neoplastic potential of these tumors is, as already mentioned, mainly based on oncogenic activation of the EGFR/RAS/RAF/MEK/ERK signaling axis [66–68]. This pathway was actively blocked by DACE in our in vitro experiments, in both human A549 lung adenocarcinoma and oncogenically transformed mouse NIH3T3 fibroblasts. In order to analyze the anti-tumor effect specifically and precisely, we used transgenic mice with lung-targeted expression of oncogenic human c-RAF kinase. The c-RAF-1-BXB mice develop within 2–4 months after birth multiple type II pneumocyte adenomas, the oncogenic potential of which is, similar to NIH3T3(v-RAF) cells [38,39] and is based on transformation by v-raf oncogene [28]. As our histological analyses showed, DACE not only suppressed the progression of RAF-mediated NSCLC in c-RAF-1-BXB transgenic mice but also reduced the amount of tumor tissue (Fig. 5), which is in good accordance with our *in vitro* studies showing a strong anti-proliferative and apoptotic action of DACE on v-raf-transformed NIH3T3(v-RAF) cells.

The in vitro studies have shown that DACE efficiently blocks activation of several signaling cascades that are related to cell survival and proliferation. However, the exact molecular mechanism by which DACE inhibits the function of kinases responsible for activation of these cascades has still to be deciphered. Nonetheless, since the nature of malignant transformation for both NIH3T3(v-RAF) tissue culture cells and the epithelial lung cells in c-RAF-1-BXB mice is similar, it is very likely that the mechanism for tumor growth inhibition and cell death in both cases is similar as well.

In conclusion, we show for the first time that the cucurbitacin B derivative DACE has a potent anti-NSCLC cell activity by interfering with the EGFR activation and its downstream signaling. In addition, its anti-tumor activity was verified, also for the first time, in a well-established c-RAF-induced lung tumor model *in vivo*. Taken together, these findings suggest that DACE is a promising lead compound for the development of an anti-lung-cancer drug.
